# Exploring the influence of problematic smartphone use on physical activity: chain mediation of Self-Control and Exercise Procrastination and moderation by Independent and Interdependent Self-Construal

**DOI:** 10.3389/fpsyg.2026.1886306

**Published:** 2026-09-03

**Authors:** Yibo Shen, Taeyang Yang, Gyuil Lee

**Affiliations:** 1 Department of Physical Education, Kyungpook National University, Daegu, Republic of Korea; 2 Department of Physical Education, Pohang Jangsung High School, Pohang, Republic of Korea

**Keywords:** chain mediation, Exercise Procrastination, Independent self-construal, Interdependent self-construal, moderation, physical activity, problematic smartphone use, Self-Control

## Abstract

**Objective:**

With smartphones deeply integrated into college students’ daily lives, Problematic Smartphone Use (PSU) may become a significant risk factor affecting Physical Activity (PA). Based on self-regulation theory and self-construal theory, this study aims to examine the relationship between PSU and college students’ PA, and further investigate the chain mediation effects of Self-Control (SC) and Exercise Procrastination (EP), as well as the moderating roles of Independent Self-Construal (IND) and Interdependent Self-Construal (INT).

**Methods:**

A large-sample questionnaire survey was conducted, collecting 1,049 valid responses from college students across four provinces in China. Established scales were used for measurement. SPSS 31.0 and AMOS 31.0 were employed for descriptive statistics, correlation analysis, and confirmatory factor analysis. Two moderated chain mediation models were constructed, with the moderating variable being IND (Model 1) and INT (Model 2), respectively. Direct, indirect, and moderating effects were examined using a bootstrap procedure with 5,000 resamples. PROCESS Model 1 was used to generate slope plots for high and low levels of IND and INT.

**Results:**

PSU negatively predicted college students’ PA. The single mediation effect of SC was not statistically significant, while the single mediation effect of EP was significant. A significant chain mediation pathway from SC to EP was identified. Self-Construal (Independent vs. Interdependent) significantly moderated the link between SC and EP in the chain mediation process.

**Conclusion:**

PSU is closely associated with insufficient PA among college students. This association may be explained by a sequential psychological-behavioral mechanism in which lower SC is linked to increased EP. Self-Construal serves as a boundary condition in this process, indicating that the association between PSU and PA may vary depending on individuals’ self-understanding.

## 1 Background

In recent years, insufficient Physical Activity (PA) has become a significant global public health concern, continuously increasing health burdens at both individual and societal levels (Strain et al., 2024; WHO, 2024). College students are at a critical transition stage from adolescence to adulthood, during which their learning patterns, lifestyle rhythms, time allocation, and health behaviors are prone to change in the university environment. Consequently, insufficient PA during this period is highly stage-specific and context-dependent (Kljajević et al., 2021). Understanding the mechanisms underlying college students’ insufficient PA and developing targeted intervention strategies has become an important topic in health behavior research.

Meanwhile, smartphones have become deeply embedded in college students’ learning, social, and recreational activities. Problematic behaviors related to smartphone use, such as overreliance and failed self-regulation, may not only affect psychological states, sleep quality, and academic performance, but also alter time allocation, attentional resources, and health behavior choices (Busch and McCarthy, 2021; Candussi et al., 2023; Pirwani and Szabo, 2024). Against this backdrop, the potential impact of Problematic Smartphone Use (PSU) on college students’ PA has gradually emerged as an important issue in research on digital lifestyles and health behaviors (Pirwani and Szabo, 2025).

Existing studies generally indicate that individuals with higher levels of PSU tend to exhibit lower levels of PA (Yang et al., 2021; Zhu et al., 2023). However, most research has focused on examining the direct relationship between the two variables. The underlying mechanisms, psychological and behavioral processes, and individual difference factors through which PSU reduces PA remain insufficiently explained (Hall and Fong, 2015; de Ridder et al., 2012). Therefore, the current study aims, on the basis of confirming the relationship between PSU and PA, to further reveal the underlying mechanisms influencing PA.

Specifically, this study targets college students and, based on self-regulation theory and self-construal theory, constructs a model incorporating chain mediation and moderation effects. It examines the mechanisms through which PSU affects PA, as well as its boundary conditions. In particular, the study focuses on the roles of Self-Control (SC) and Exercise Procrastination (EP) in the pathway from PSU to PA, and further tests whether Independent Self-Construal (IND) and Interdependent Self-Construal (INT) alter this process. The findings will help deepen the understanding of how digital lifestyles influence college students’ PA and provide theoretical guidance for interventions promoting PA and reducing PSU. Theoretically, this study extends self-regulation theory by distinguishing between general self-regulatory capacity (SC) and exercise-specific behavioral execution failure (EP), and refines self-construal theory by showing that IND and INT function as boundary conditions in the self-regulation process rather than merely as direct predictors of health behavior.

### 1.1 Direct effect of PSU on PA

From the perspective of self-regulation theory, PA is a goal-directed behavior that requires planning, initiation, and persistence, whereas PSU tends to attract individuals with immediate gratification and short-term entertainment (Hall and Fong, 2015). PSU is not merely reflected in longer smartphone usage time, but rather in tendencies such as difficulty controlling use, overreliance, frequent checking, and impairment of daily functioning (Busch and McCarthy, 2021; Candussi et al., 2023). Due to smartphones’ high accessibility, instant feedback, and continuous stimulation, they can constantly occupy college students’ attentional resources and fragmented time, thereby reducing investment in PA (Pirwani and Szabo, 2025).

In contrast, PA typically requires time allocation, behavioral preparation, and sustained effort, with health benefits often realized as long-term rewards. Therefore, when college students exhibit higher levels of PSU, they are more likely to prioritize the immediate gratification of smartphone use over engaging in PA. Previous studies have also shown an overall negative relationship between PSU and PA, indicating that higher levels of PSU are generally associated with lower levels of PA (Yang et al., 2021; Zhu et al., 2023; Pirwani and Szabo, 2024). Accordingly, PSU may constitute an important risk factor for reduced participation in PA among college students.

*H1*: PSU significantly negatively predicts PA (path a).

### 1.2 Mediating role of SC

From a self-regulation perspective, PSU may influence PA by weakening individuals’ SC. SC is a critical psychological resource that enables individuals to resist immediate temptations, inhibit impulsive responses, and persist toward long-term goals (de Ridder et al., 2012). However, PSU, characterized by immediate feedback, high-frequency stimulation, and continuous attentional capture, can lead to overreliance on smartphones and reduce control over goal-directed behaviors (Busch and McCarthy, 2021; Candussi et al., 2023). Prior studies indicate that individuals with lower SC are more prone to PSU, which can further deplete attentional and behavioral control abilities (Jiang and Zhao, 2016; Fabio et al., 2022). Therefore, higher levels of PSU are associated with lower SC among college students.

Reduced SC may further lead to lower levels of PA. Engaging in PA requires planning, initiation, and sustained effort, which are not solely determined by awareness of exercise benefits (Hall and Fong, 2015). In daily college life, participating in PA often requires resisting smartphone entertainment, sedentary study habits, fatigue, and other immediate temptations. Previous studies have shown that SC is closely related to health behaviors, including exercise, healthy eating, and sleep, and higher SC facilitates the initiation and maintenance of PA (Boat and Cooper, 2019; Andrade and Hoyle, 2023). Therefore, when PSU impairs SC, college students may find it more difficult to persist in PA, resulting in reduced levels.

*H2*: SC mediates the relationship between PSU and PA (path bf).

### 1.3 Mediating role of EP

From a behavioral execution perspective, PSU may reduce PA by increasing EP. EP refers to the tendency to delay, avoid, or fail to initiate exercise behaviors, even when individuals recognize the importance of exercise and intend to engage in it. Although EP is conceptually related to exercise intention, it differs from low intention because it emphasizes the failure to translate existing exercise intention or awareness into timely behavioral execution (Kelly and Walton, 2021; Miao et al., 2024). PSU, with its immediate rewards, low-cost entertainment, and continuous feedback, can lead individuals to prioritize smartphone use over exercise, which requires physical effort, time management, and self-regulation (Busch and McCarthy, 2021). Studies indicate a significant positive correlation between PSU and procrastination behaviors, suggesting that excessive smartphone use strengthens the tendency to postpone tasks and avoid action (Chen and Lyu, 2024). Accordingly, higher levels of PSU are associated with higher levels of EP.

Increased EP may further reduce PA. Engagement in PA depends not only on exercise intention but also on translating that intention into timely action and sustaining it in daily life. EP reflects the gap between exercise cognition, intention, and actual behavior execution (Kelly and Walton, 2021). Studies have found that even when controlling for general procrastination and exercise intention, EP still significantly predicts lower levels of PA, indicating its role as a proximal behavioral mechanism explaining insufficient exercise (Kelly and Walton, 2021). Therefore, when college students are more prone to delay exercise due to PSU, planned PA is often replaced by smartphone entertainment, social browsing, or information checking, ultimately leading to reduced actual PA.

*H3*: EP mediates the relationship between PSU and PA (path ed).

### 1.4 Chain mediation of SC and EP

From the perspective of self-regulation theory, PSU may reduce PA through a sequential process: declining SC leading to increased EP. SC is a generalized psychological resource reflecting the ability to inhibit impulses, resist temptations, and persist toward long-term goals (de Ridder et al., 2012). In contrast, EP is a situational behavioral manifestation, reflecting delays, avoidance, or failures in initiating and executing exercise (Kelly and Walton, 2021; Miao et al., 2024). Therefore, SC can be viewed as a psychological self-regulation resource, while EP represents the behavioral consequence of depleted self-regulation resources in the context of PA.

The immediate gratification and continuous stimulation characteristics of PSU can weaken attentional and behavioral control, making it difficult for college students to disengage from smartphone use and allocate behavioral resources to long-term health goals such as PA (Jiang and Zhao, 2016; Fabio et al., 2022). When SC is reduced, individuals are more likely to delay, avoid, or fail to execute exercise behaviors, leading to higher EP (Kelly and Walton, 2021). Subsequently, EP causes planned PA to be continually postponed, reducing actual exercise frequency and duration, ultimately resulting in decreased PA.

Therefore, the effect of PSU on PA may not occur through a single pathway, but rather through a continuous process linking psychological resource depletion to behavioral execution failure. Previous studies also suggest a close connection among PSU, SC, and EP, indicating that these three factors jointly constitute an important psychological-behavioral mechanism affecting PA (He et al., 2025).

*H4*: SC and EP sequentially mediate the relationship between PSU and PA (path bcd).

### 1.5 Moderating role of IND and INT

According to self-construal theory, individuals’ understanding of the self, goals, and social relationships influences goal selection, behavioral regulation, and coping strategies. Self-construal generally includes IND and INT. The former emphasizes personal goals, autonomous choices, and self-consistency, while the latter emphasizes social relationships, role responsibilities, and contextual adaptation (Markus and Kitayama, 1991; Singelis, 1994; Cross et al., 2011). In the context of PA, the effectiveness of SC in translating into exercise execution may be influenced by self-construal. Therefore, self-construal may not only affect EP but also moderate the relationship between SC and EP, further affecting the indirect pathway from PSU to PA via SC and EP.

Individuals with high IND generally prioritize personal goals, autonomous choice, and internal standards (Markus and Kitayama, 1991; Singelis, 1994). In exercise contexts, these individuals may more easily view PA as part of personal health goals and self-management, reducing dependence on general SC. In contrast, those with low IND may lack stable autonomous goals and rely more on SC to reduce EP when facing smartphone entertainment and behavioral inertia. Therefore, IND may influence EP and moderate the link between SC and EP.

Individuals with high INT generally focus on social relationships, group norms, and situational responsibilities (Markus and Kitayama, 1991; Cross et al., 2011). In exercise contexts, their PA may be more influenced by peer support, social expectations, and collective atmosphere, potentially altering the effect of SC on EP. Conversely, individuals with low INT rely less on external relationships and contextual support and depend more on personal SC to overcome exercise initiation difficulties. Therefore, INT may affect EP and moderate the relationship between SC and EP.

*H5*: IND moderates the relationship between SC and EP (path h).

*H6*: INT moderates the relationship between SC and EP (path h).

*H7*: IND moderates the chain mediation path from PSU through SC and EP to PA (path bchd).

*H8*: INT moderates the chain mediation path from PSU through SC and EP to PA (path bchd).

### 1.6 Research model and path coding

**Figure 1:**
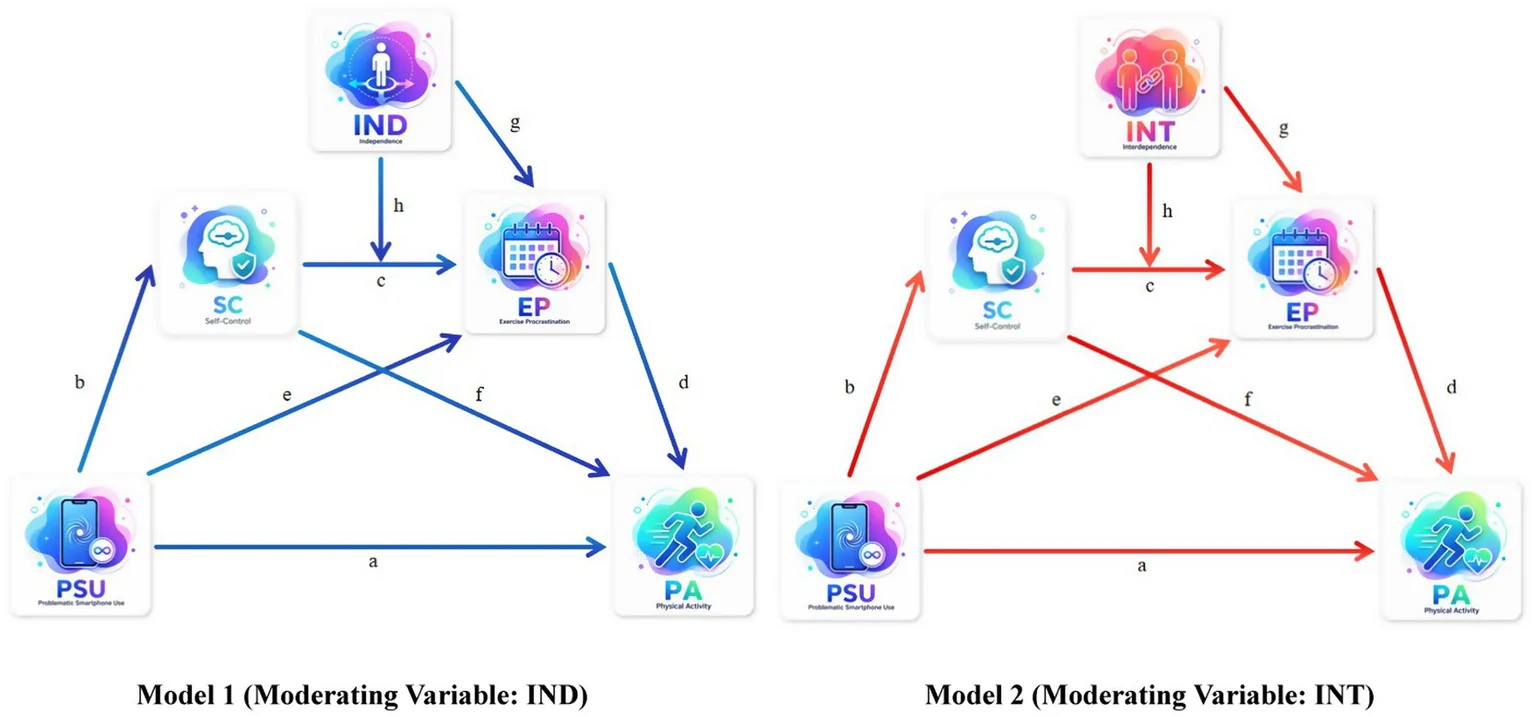
Research model. PSU = problematic smartphone use; SC = self-control; EP = exercise procrastination; IND = independent self-construal; INT = interdependent self-construal; PA = physical activity. Path a represents the direct path from PSU to PA; path b represents PSU → SC; path c represents SC → EP; path d represents EP → PA; path e represents PSU → EP; path f represents SC → PA; path g represents IND/INT → EP; and path h represents the interaction effect of SC × IND/SC × INT on EP. Based on these path labels, the indirect effect through SC was calculated as *b* × *f*, the indirect effect through EP as *e* × *d*, the serial mediation effect as *b* × *c* × *d*, and the moderated serial mediation effect as *b* × *c* × *h* × *d*.

Based on the above theoretical analysis and literature review, this study constructs the research model shown in Figure 1, aiming to explore the mechanisms through which PSU affects college students’ PA, examine the single and chain mediation effects of SC and EP, and further test the moderating roles of IND and INT on the relationship between SC and EP as well as the chain mediation process.

## 2 Methods

### 2.1 Participants

This study employed a convenience sampling method (Golzar et al., 2022), targeting college students enrolled in universities across Shandong, Henan, Sichuan, and Liaoning provinces in China. The questionnaire was distributed through an online platform and disseminated with the assistance of university instructors via class groups, course groups, or student management groups. All participants completed the questionnaire voluntarily. The first page of the questionnaire explained the research purpose, instructions for completion, principles of anonymity and confidentiality, and informed participants that they could withdraw from the study at any time. Only participants who provided informed consent were allowed to proceed with the questionnaire.

After collection, the researchers screened the data based on completeness, response time, response patterns, and duplicate responses. A total of 1,124 questionnaires were collected, and 75 invalid responses were excluded, yielding a final valid sample of 1,049, with an effective response rate of 93.3%.

Among the valid sample, 505 participants were male (48.1%) and 544 were female (51.9%). The distribution by grade level was as follows: third-year students were the largest group with 389 participants (37.1%), followed by second-year students with 271 (25.8%), fourth-year students with 178 (17.0%), first-year students with 175 (16.7%), and fifth-year or above with 36 (3.4%). By major, participants were primarily from the humanities and social sciences (344, 32.8%), followed by natural sciences and engineering (187, 17.8%), physical education and arts (175, 16.7%), health sciences (160, 15.3%), education (118, 11.2%), and agricultural sciences (65, 6.2%). Regarding subjective health status, 586 participants (55.9%) rated themselves as healthy, 283 (26.9%) as relatively poor, and 180 (17.2%) as average. Detailed demographic characteristics are presented in Table 1.

**Table 1:** Demographic characteristics of participants.

Variable	Category	*n* (%)	Variable	Category	*n* (%)
Gender	Male	505 (48.1%)	Grade	Freshman	175 (16.7%)
	Female	544 (51.9%)		Sophomore	271 (25.8%)
Major	Humanities and Social Sciences	344 (32.8%)		Junior	389 (37.1%)
	Natural Sciences and Engineering	187 (17.8%)		Senior	178 (17.0%)
	Physical Education and Arts	175 (16.7%)		Fifth year or above	36 (3.4%)
	Health Sciences	160 (15.3%)	Health Status	Poor	283 (26.9%)
	Education	118 (11.2%)		Fair	180 (17.2%)
	Agricultural Sciences	65 (6.2%)		Good	586 (55.9%)

### 2.2 Measures

The core variables of this study included Problematic Smartphone Use (PSU), Self-Control (SC), Exercise Procrastination (EP), Physical Activity (PA), Independent Self-Construal (IND) and Interdependent Self-Construal (INT). Except for PA, which was calculated using the IPAQ-SF to obtain total PA, all other variables were measured using Likert-type scales, with higher scores indicating higher levels of the corresponding construct.

*Problematic Smartphone Use*: The concept of PSU was measured using the Smartphone Addiction Scale-Short Version (SAS-SV) developed by Kwon et al. (2013) and validated in Chinese by Luk et al. (2018). The scale is unidimensional, consisting of 10 items scored on a 6-point Likert scale, primarily assessing control failure, excessive use, and impairment of daily functioning in smartphone use. In this study, Cronbach’s *α* = 0.974.

*Self-Control*: SC was assessed using the Brief Self-Control Scale (BSCS) developed by Tangney et al. (2004) and validated in Chinese college student samples by Fung et al. (2020). The scale is unidimensional, consisting of 11 items scored on a 5-point Likert scale, assessing impulse control, self-regulation, and long-term goal orientation. Cronbach’s *α* = 0.965.

*Exercise Procrastination*: EP was measured using the Irrational Procrastination Scale (IPS) developed by Steel (2010) and validated in Chinese by Shaw and Zhang (2021). To fit the exercise context of the present study, the original items were semantically adapted by replacing general task-related expressions with exercise-related expressions while retaining the original conceptual meaning of irrational delay and behavioral avoidance. For example, items referring to delaying tasks were revised to reflect delaying planned exercise or failing to initiate intended exercise behavior. Following the semantic adaptation, the adapted items were independently reviewed by two experts, including one professor in Department of Physical Education and one lecturer with several years of research experience in sport and exercise psychology. The expert review focused on (1) consistency with the conceptual meaning of the original items, (2) relevance to exercise procrastination, (3) clarity and comprehensibility of the wording, and (4) appropriateness for the exercise context. Minor wording adjustments were made based on the experts’ feedback, and items requiring refinement were discussed until agreement was reached. Because this procedure involved the semantic and contextual adaptation of an established measure rather than the development of a new instrument, the expert review was qualitative in nature and no quantitative Content Validity Index (CVI) was calculated. The adapted scale remained unidimensional and consisted of 9 items rated on a 5-point Likert scale, with higher scores indicating a stronger tendency to delay, avoid, or fail to execute exercise behaviors. In the present sample, the adapted scale demonstrated good internal consistency and construct validity, with Cronbach’s *α* = 0.947, standardized factor loadings above 0.70, CR = 0.948, and AVE = 0.668.

*Self-Construal*: Self-Construal was measured using the Self-Construal Scale (SCS) developed by Singelis (1994) and revised in Chinese by Wang et al. (2008). The scale includes two dimensions: IND (12 items) and INT (12 items), totaling 24 items scored on a 7-point Likert scale. IND reflects the tendency to emphasize autonomy, uniqueness, and personal goals, while INT reflects the tendency to value interpersonal relationships, group belonging, and social coordination. Cronbach’s *α* was 0.975 for IND and 0.973 for INT.

*Physical Activity*: PA was assessed using the International Physical Activity Questionnaire-Short Form (IPAQ-SF), which was developed for population-based physical activity surveillance and has been widely used to obtain comparable estimates of physical activity across different populations (Craig et al., 2003). The Chinese version validated by Wang et al. (2013) was used in the present study. Although the IPAQ-SF may overestimate moderate-to-vigorous physical activity in populations with high sedentary behavior, it remains one of the most widely used and internationally recommended instruments for large-scale epidemiological studies and population surveillance. The IPAQ-SF consists of 7 items assessing the frequency and duration of vigorous-intensity activity, moderate-intensity activity, walking, and sedentary behavior during the past 7 days. Following the standard IPAQ-SF scoring protocol, total PA was calculated as MET-min/week using the weighted sum of vigorous activity, moderate activity, and walking. The sedentary behavior item was not included in the total PA score because it is used as a separate indicator of sitting time rather than as a component of total energy expenditure in the IPAQ-SF scoring protocol. Because PA expressed in MET-min/week differs substantially in scale and unit from the Likert-type psychological variables included in the structural model, the total PA score was transformed into a standardized *z*-score before subsequent analyses. This standardization was used to improve the comparability of coefficients and model estimation stability when variables with different measurement units were analyzed together (Schielzeth, 2010).

Total PA calculation formula:


$$\begin{array}{c} \text{Total}\;\;\mathrm{PA}=8.0\times (\mathrm{VP}A_{d}\times \mathrm{VP}A_{m})+4.0(\mathrm{MP}A_{d}\times \mathrm{MP}A_{m})\\ +3.3(W_{d}\times W_{m}) \end{array}$$

Units: Met – min/week

*VPA_d_* = day of vigorous PA; *VPA_m_* = minutes/day of vigorous PA

*MPA_d_* = day of moderate PA; *MPA_m_* = miutes/day of moderate PA

*W_d_* = day of walking; *W_m_* = minutes/day of walkig

Standardized calculation formula for Total PA:


$$Z_{\mathrm{PA}}=\frac{\text{Total}\;\;\mathrm{PA}-M}{\mathrm{SD}}$$

*M* = Mea of Total PA; *SD* = standard deviation of Total PA

All measurement instruments used in this study were based on established scales and were semantically adapted to the context of Chinese college students and exercise behavior. Except for PA, which was calculated in MET-min/week according to the IPAQ-SF, all other psychological variables demonstrated high internal consistency, with Cronbach’s α coefficients ranging from 0.947 to 0.975.

### 2.3 Data analysis

Various statistical methods were employed to ensure the reliability and validity of the measurement model and to systematically examine the significance of combined direct, mediating, and moderating effects (significance threshold set at *p* < 0.05). At the initial stage, the data were cleaned, screened, and standardized to unify measurement scales and improve model estimation stability. It should be noted that the term “combined effects” in this study does not refer to a combination of random and fixed effects in multilevel linear models, but rather to the simultaneous presence of direct effects, mediating effects, and moderating effects within the same theoretical model.

First, to detect potential common method bias (CMB), a single-factor confirmatory factor analysis (CFA) was conducted using AMOS 31.0, with all measurement items loaded onto a single latent factor to construct a single-factor model, thereby examining whether substantial common method bias existed in the data.

Second, for measurement model validation, AMOS 31.0 was used to perform CFA on the latent variables of PSU, SC, EP, and Self-Construal (IND and INT) to assess construct validity. Additionally, Cronbach’s *α*, composite reliability (CR), and average variance extracted (AVE) were used to evaluate internal consistency reliability, convergent validity, and discriminant validity of each scale.

Next, in testing the combined effects, AmosEstimandVB programming combined with the Bootstrap method (5,000 resamples) was used to obtain bias-corrected confidence intervals for the path coefficients of direct, mediating, and moderating effects. Effects were considered statistically significant if the confidence interval did not include zero.

For the moderation analysis, the interaction terms were constructed using an observed composite-score product approach. Scale-level composite scores for SC and the corresponding moderator (IND or INT) were mean-centered before multiplication. Specifically, SC × IND was computed from the mean-centered SC and IND scores for Model 1, whereas SC × INT was computed from the mean-centered SC and INT scores for Model 2. In each model, the centered lower-order terms and the corresponding product term were entered simultaneously into the AMOS structural model. Mean-centering was used to reduce nonessential multicollinearity and to facilitate interpretation of the lower-order effects in the presence of the interaction term (Aiken et al., 1991; Hayes, 2017).

Finally, PROCESS Model 1 was employed for simple slope analysis, and simple slope plots were generated for high and low levels of IND and INT, visually presenting the direction and strength of their moderating effects on the relationship between SC and EP.

## 3 Results

### 3.1 Common method Bias and model fit

To reduce potential systematic errors arising from a single source of measurement, procedural controls were implemented in the questionnaire design, including anonymous responses, randomized item presentation, and cross-ordering of different constructs. At the statistical level, common method bias (CMB) was assessed through single-factor confirmatory factor analysis (CFA) and comparison with the full multi-factor model (Podsakoff et al., 2003).

The results showed that the single-factor model for Model 1 yielded the following fit indices: *χ^2^* = 29,739.521; df = 860; *χ^2^/df* = 34.581; GFI = 0.203; AGFI = 0.123; CFI = 0.401; NFI = 0.395; TLI = 0.371; RMSEA = 0.179. For Model 2, the single-factor model fit indices were: *χ^2^* = 29,813.598; df = 860; *χ^2^/df* = 34.667; GFI = 0.203; AGFI = 0.123; CFI = 0.403; NFI = 0.396; TLI = 0.373; RMSEA = 0.179. Overall, the single-factor models of Model 1 and Model 2 exhibited substantially poorer fit compared to the full multi-factor models (Table 2), suggesting that severe common method bias was unlikely to substantially threaten the validity of the findings (Podsakoff et al., 2003). However, because all variables were collected through self-report questionnaires at the same time point, the possibility of residual common method bias cannot be completely ruled out. Therefore, the results should be interpreted with appropriate caution, and future studies are encouraged to combine self-report data with objective behavioral indicators or multi-source data.

**Table 2:** Model fit.

Variable/Model	Items (*n*)	*χ^2^*	*df*	*χ^2^/df*	GFI	AGFI	NFI	RMSEA	Cronbach’s *α*
Model 1	43	941.255	490	1.921	0.952	0.945	0.972	0.030	–
Model 2	43	950.818	490	1.940	0.951	0.944	0.972	0.030	–
PSU	10	55.534	35	1.587	0.990	0.984	0.996	0.024	0.974
SC	11	67.627	44	1.537	0.989	0.983	0.994	0.023	0.965
EP	9	44.822	27	1.660	0.991	0.984	0.994	0.025	0.947
IND	12	85.451	54	1.582	0.986	0.980	0.994	0.024	0.975
INT	12	73.437	54	1.360	0.989	0.984	0.995	0.019	0.973

The criteria for model fit were χ^2^/df <3; GFI, AGFI, CFI, NFI, and TLI >0.90; RMSEA <0.06, Cronbach’s *α* > 0.70.

Model 1 (Moderating Variable: IND), Model 2 (Moderating Variable: INT). PSU, Problematic Smartphone Use; SC, Self-Control; EP, Exercise Procrastination; IND, Independent Self-Construal; INT, Interdependent Self-Construal.

Table 2 shows that the overall model fit indices indicate good alignment between the theoretical model and the sample data, meeting the recommended standards in the literature (Hu and Bentler, 1999). The fit indices of each measurement instrument (latent variable) were also within the ideal range, further supporting the construct validity of the scales and the full model, providing a solid foundation for subsequent analyses of chain mediation and moderating effects (Kline, 2023).

### 3.2 Confirmatory factor analysis

Confirmatory factor analysis (CFA) was conducted using AMOS 31.0 for the five core latent variables, and SMC, CR, and AVE were calculated to examine the convergent validity of each latent variable.

As shown in Table 3, all items for the latent variables were significant (*p* < 0.001) and had standardized factor loadings (Std.) greater than 0.70, indicating that the items effectively reflected the corresponding latent variables. Composite reliability (CR) ranged from 0.948 to 0.976, exceeding the recommended threshold of 0.60. The average variance extracted (AVE) ranged from 0.668 to 0.790, surpassing the 0.50 criterion, indicating good convergent validity of the scales. The squared multiple correlations (SMC) of items were greater than 0.50, demonstrating strong explanatory power and indicator stability.

**Table 3:** Confirmatory factor analysis results.

Latent variable	Items	Unstd.	S. E.	*z*	*p*	Std.	SMC	CR	AVE
PSU	PSU1	1.000				0.981	0.962	0.974	0.790
	PSU2	0.908	0.016	58.551	^***^	0.891	0.794		
	PSU3	0.888	0.017	53.509	^***^	0.871	0.759		
	PSU4	0.896	0.016	55.576	^***^	0.880	0.774		
	PSU5	0.893	0.016	54.819	^***^	0.877	0.769		
	PSU6	0.889	0.017	53.774	^***^	0.872	0.760		
	PSU7	0.895	0.016	55.235	^***^	0.878	0.771		
	PSU8	0.896	0.016	55.491	^***^	0.879	0.773		
	PSU9	0.890	0.017	53.960	^***^	0.873	0.762		
	PSU10	0.896	0.016	55.505	^***^	0.880	0.774		
SC	SC1	1.000				0.971	0.943	0.965	0.714
	SC2	0.854	0.019	44.275	^***^	0.829	0.687		
	SC3	0.866	0.019	45.982	^***^	0.840	0.706		
	SC4	0.854	0.019	44.303	^***^	0.829	0.687		
	SC5	0.861	0.019	45.311	^***^	0.836	0.699		
	SC6	0.857	0.019	44.610	^***^	0.831	0.691		
	SC7	0.878	0.018	47.986	^***^	0.852	0.726		
	SC8	0.851	0.019	43.769	^***^	0.826	0.682		
	SC9	0.855	0.019	44.336	^***^	0.830	0.689		
	SC10	0.843	0.020	42.658	^***^	0.818	0.669		
	SC11	0.846	0.020	43.069	^***^	0.821	0.674		
EP	EP1	1.000				0.918	0.843	0.948	0.668
	EP2	0.925	0.023	41.195	^***^	0.849	0.721		
	EP3	0.893	0.024	38.012	^***^	0.819	0.671		
	EP4	0.850	0.025	34.378	^***^	0.780	0.608		
	EP5	0.855	0.025	34.807	^***^	0.785	0.616		
	EP6	0.853	0.025	34.680	^***^	0.783	0.613		
	EP7	0.859	0.025	35.117	^***^	0.788	0.621		
	EP8	0.870	0.024	36.046	^***^	0.799	0.638		
	EP9	0.901	0.023	38.802	^***^	0.827	0.684		
IND	IND1	1.000				0.983	0.966	0.975	0.765
	IND2	0.880	0.017	52.413	^***^	0.865	0.748		
	IND3	0.892	0.016	55.180	^***^	0.877	0.769		
	IND4	0.885	0.017	53.569	^***^	0.870	0.757		
	IND5	0.878	0.017	52.024	^***^	0.863	0.745		
	IND6	0.892	0.016	55.036	^***^	0.876	0.767		
	IND7	0.876	0.017	51.570	^***^	0.861	0.741		
	IND8	0.865	0.018	49.377	^***^	0.850	0.723		
	IND9	0.862	0.018	48.779	^***^	0.847	0.717		
	IND10	0.884	0.017	53.248	^***^	0.869	0.755		
	IND11	0.872	0.017	50.831	^***^	0.857	0.734		
	IND12	0.883	0.017	52.970	^***^	0.867	0.752		
INT	INT1	1.000				0.985	0.970	0.976	0.769
	INT2	0.891	0.016	55.914	^***^	0.878	0.771		
	INT3	0.887	0.016	54.874	^***^	0.874	0.764		
	INT4	0.871	0.017	51.533	^***^	0.859	0.738		
	INT5	0.876	0.017	52.423	^***^	0.863	0.745		
	INT6	0.873	0.017	51.903	^***^	0.860	0.740		
	INT7	0.879	0.017	53.066	^***^	0.866	0.750		
	INT8	0.873	0.017	51.926	^***^	0.861	0.741		
	INT9	0.882	0.016	53.775	^***^	0.869	0.755		
	INT10	0.877	0.017	52.650	^***^	0.864	0.746		
	INT11	0.881	0.017	53.514	^***^	0.868	0.753		
	INT12	0.880	0.017	53.386	^***^	0.867	0.752		

***p < 0.001.

PSU, Problematic Smartphone Use; SC, Self-Control; EP, Exercise Procrastination; IND, Independent Self-Construal; INT, Interdependent Self-Construal.

All instruments met internationally recognized discriminant validity criteria, including standardized factor loadings greater than 0.70, CR above 0.60, and AVE and SMC exceeding 0.50 (Fornell and Larcker, 1981). Therefore, the measurement instruments demonstrated good reliability and convergent validity, with high measurement stability and explanatory power.

### 3.3 Correlation and descriptive statistics

To further examine the relationships among the latent variables and the characteristics of the data distribution, correlation and descriptive statistical analyses were conducted for all core variables. As shown in Table 4, all variables were significantly correlated (*p* < 0.01), and no excessively high correlations were observed, indicating that severe multicollinearity was not present (Hair et al., 2019). In addition, auxiliary collinearity diagnostics showed that the VIF values for SC and EP were below the commonly accepted threshold and tolerance values were above 0.10, suggesting that the non-significant SC → PA path was unlikely to be mainly attributable to multicollinearity. Rather, the result indicates that after EP was included in the model, the independent contribution of SC to PA became limited. In terms of discriminant validity, the square roots of the AVE for each latent variable were greater than their correlations with other variables, supporting good discriminant validity (Fornell and Larcker, 1981). At the structural level, SC and EP were also treated as theoretically distinct constructs: SC reflects a general psychological self-regulation resource, whereas EP represents a more proximal behavioral tendency to delay or avoid exercise. The non-significant SC → PA path, together with the significant SC → EP → PA serial pathway, suggests that SC may be related to PA primarily through exercise-specific behavioral execution rather than through a direct effect on PA.

**Table 4:** Correlations, descriptive statistics, and square roots of AVEs.

Variable	*m*	SD	Skew	Kurtosis	AVE	PA	PSU	SC	EP	IND	INT
PA	5838.55	2588.551	0.571	0.030	–	–					
PSU	3.95	1.392	−0.188	−1.086	0.790	−0.294^**^	***0.889***				
SC	3.61	1.147	−0.392	−1.268	0.714	0.176^**^	−0.442^**^	***0.845***			
EP	4.15	0.823	−1.927	3.807	0.668	−0.380^**^	0.500^**^	−0.303^**^	***0.817***		
IND	4.39	1.792	−0.161	−1.383	0.765	0.010	−0.356^**^	0.403^**^	−0.146^**^	***0.875***	
INT	4.67	1.824	−0.280	−1.367	0.769	0.017	−0.371^**^	0.402^**^	−0.149^**^	0.506^**^	***0.877***

^**^p < 0.01; Italicized bold values on the diagonal represent the square roots of AVEs.

Regarding descriptive statistics, the means and standard deviations of all variables were within reasonable ranges, and skewness and kurtosis did not exceed commonly used thresholds, suggesting that the overall data distribution largely conformed to the assumption of normality (West et al., 1995).

Overall, the results of correlation, discriminant validity, and data distribution indicate good data quality, providing a solid foundation for subsequent analyses of chain mediation and moderating effects.

### 3.4 Analysis of combined effects model

Structural equation modeling (SEM) was conducted using AMOS, and the Bootstrap method with 5,000 resamples was applied to test direct, mediating, and moderating effects. To examine the moderating effects, two interaction terms, SC × IND and SC × INT, were created and incorporated into Model 1 and Model 2, respectively.

#### 3.4.1 Path coefficients

**Figure 2:**
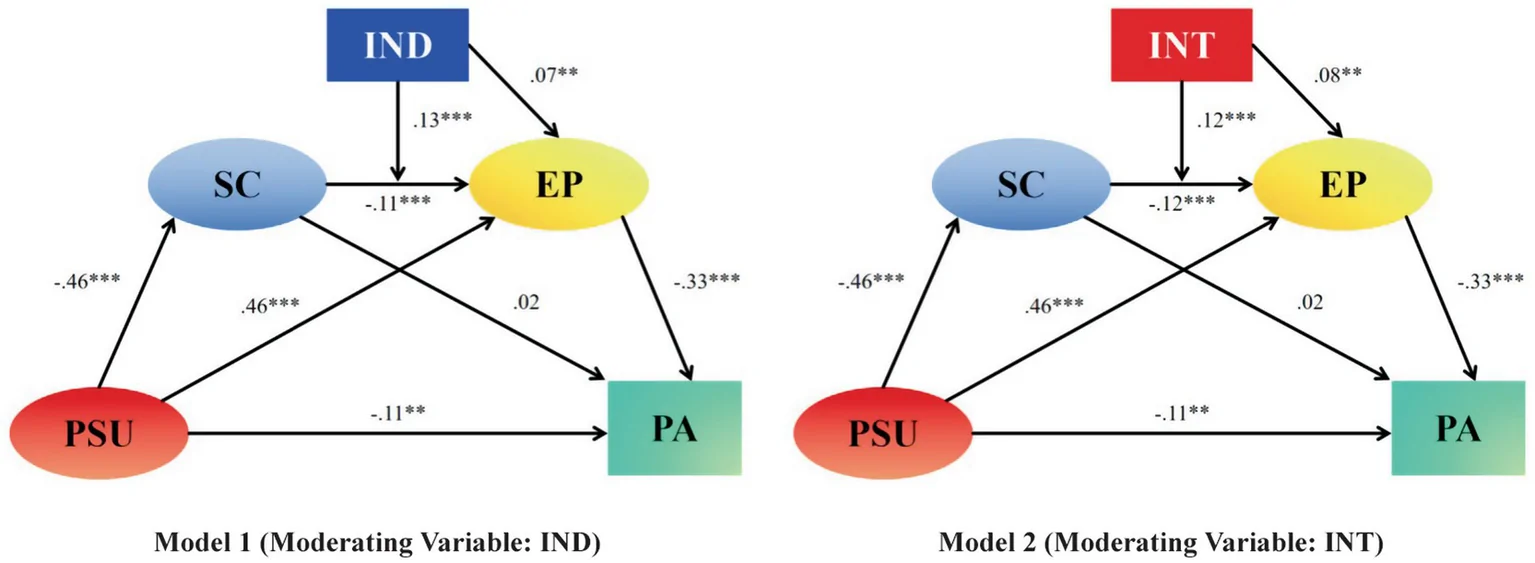
Model and path coefficients. **p < 0.01, ***p < 0.001. Standardized regression weights (*β*). PSU = Problematic Smartphone Use; SC = Self-Control; EP = Exercise Procrastination; IND = Independent Self-Construal; INT = Interdependent Self-Construal; PA = Physical Activity. Effect a: PSU → PA; Effect b: PSU → SC; Effect c: SC → EP; Effect d: EP → PA; Effect e: PSU → EP; Effect f: SC → PA; Effect g: IND/INT → EP; Effect h: SC × IND/SC × INT → EP.

Figure 2 presents the standardized coefficients for all paths in the models. To test the path relationships and moderating effects, Model 1 (with IND as the moderating variable) and Model 2 (with INT as the moderating variable) were analyzed. The results are as follows:

In Model 1, the path from PSU to PA (path a) was *β* = −0.11, *p* < 0.01; the path from PSU to SC (path b) was *β* = −0.46, *p* < 0.001; the path from SC to EP (path c) was *β* = −0.11, *p* < 0.001; the path from EP to PA (path d) was *β* = −0.33, *p* < 0.001; the path from PSU to EP (path e) was *β* = 0.46, *p* < 0.001; the path from SC to PA (path f) was *β* = 0.02, *p* > 0.05; the path from IND to EP (path g) was *β* = 0.07, *p* < 0.01; and the interaction term (SC × IND) on EP (path h) was *β* = 0.13, *p* < 0.001.

In Model 2, the path from PSU to PA (path a) was *β* = −0.11, *p* < 0.01; the path from PSU to SC (path b) was *β* = −0.46, *p* < 0.001; the path from SC to EP (path c) was *β* = −0.12, *p* < 0.001; the path from EP to PA (path d) was *β* = −0.33, *p* < 0.001; the path from PSU to EP (path e) was *β* = 0.46, *p* < 0.001; the path from SC to PA (path f) was *β* = 0.02, *p* > 0.05; the path from INT to EP (path g) was *β* = 0.08, *p* < 0.01; and the interaction term (SC × INT) on EP (path h) was *β* = 0.12, *p* < 0.001.

#### 3.4.2 Effect analysis of model 1

In AMOS, direct, mediating, and moderating effects were coded as paths a–h. An AmosEstimandVB programming script was created, and instructions were implemented corresponding to the effect types for hypotheses H1–H8. Further, estimates and tests were conducted separately for Model 1 (with IND as the moderating variable) and Model 2 (with INT as the moderating variable), yielding the results of direct, mediating, and moderating effects shown in Tables 5 and 6.

**Table 5:** Model 1 (moderating variable: IND).

Hypothesis/Effect		*β*	Product of		95% CI		*p*
			S. E.	*z*	Lower	Upper	
H1	Direct effect (a)	−0.117^**^	0.041	−2.854	−0.200	−0.035	0.0038
H2	Indirect effect (bf)	−0.011	0.014	−0.786	−0.039	0.016	0.4438
H3	Indirect effect (ed)	−0.154^***^	0.027	−5.704	−0.212	−0.106	0.0008
H4	Serial mediation effect (bcd)	−0.017^***^	0.006	−2.833	−0.032	−0.007	0.0007
H5	Direct effect (g)	0.036	0.022	1.636	−0.007	0.077	0.1096
	Moderation effect (h)	0.131^***^	0.034	3.853	0.063	0.198	0.0009
H7	Moderated mediation effect (bchd)	−0.002^***^	0.001	−2.000	−0.005	−0.001	0.0004
	Total	−0.134^**^	0.049	−2.735	−0.232	−0.042	0.0051

Path a = PSU → PA; path b = PSU → SC; path c = SC → EP; path d = EP → PA; path e = PSU → EP; path f = SC → PA; path g = IND → EP; path h = SC × IND → EP. The indirect effect through SC was calculated as b × f; the indirect effect through EP was calculated as e × d; the serial mediation effect was calculated as b × c × d; and the moderated serial mediation effect was calculated as b × c × h × d. Total effect = a + bf + ed + bcd + g + h + bchd.

^*^p < 0.05, ^**^p < 0.01, ^***^p < 0.001. User-defined estimand effect estimates + bootstrap inference.

**Table 6:** Model 2 (moderating variable: INT).

Hypothesis/Effect		*β*	Product of		95% CI		*p*
			S. E.	*z*	Lower	Upper	
H1	Direct effect (a)	−0.117^**^	0.041	−2.854	−0.200	−0.035	0.0038
H2	Indirect effect (bf)	−0.011	0.014	−0.786	−0.039	0.016	0.4435
H3	Indirect effect (ed)	−0.154^***^	0.027	−5.704	−0.212	−0.105	0.0008
H4	Serial mediation effect (bcd)	−0.018^***^	0.006	−3.000	−0.033	−0.007	0.0006
H6	Direct effect (g)	0.041^*^	0.020	2.050	0.002	0.080	0.0431
	Moderation effect (h)	0.119^***^	0.032	3.719	0.060	0.186	0.0008
H8	Moderated mediation effect (bchd)	−0.002^***^	0.001	−2.000	−0.005	−0.001	0.0006
	Total	−0.142^**^	0.049	−2.898	−0.237	−0.043	0.0013

Path a = PSU → PA; path b = PSU → SC; path c = SC → EP; path d = EP → PA; path e = PSU → EP; path f = SC → PA; path g = INT → EP; path h = SC × INT → EP. The indirect effect through SC was calculated as b × f; the indirect effect through EP was calculated as e × d; the serial mediation effect was calculated as b × c × d; and the moderated serial mediation effect was calculated as b × c × h × d. Total effect = a + bf + ed + bcd + g + h + bchd.

^*^p < 0.05, ^**^p < 0.01, ^***^p < 0.001. User-defined estimand effect estimates + bootstrap inference.

Model 1 showed a total effect of −0.134, with SE = 0.049. The *z* value, calculated as Mean/SE, was −2.735, exceeding the 1.96 threshold, and the 95% confidence interval [−0.232, −0.042] did not include zero, indicating that the total effect was significant.

For H1, the direct effect path (effect “a”) had *β* = −0.117, SE = 0.041, *z* = −2.854, 95% CI [−0.200, −0.035], which did not include zero; the *z* value was significant, supporting H1.

For H2, the mediating effect path (effect “bf”) had *β* = −0.011, SE = 0.014, *z* = −0.786, 95% CI [−0.039, 0.016], indicating that H2 was not supported. Given the large sample size of this study (*n* = 1,049) and the use of 5,000 bootstrap resamples, the non-significant indirect effect is more likely attributable to the very small effect size and the non-significant SC → PA path, rather than to insufficient statistical power alone.

For H3, the mediating effect path (effect “ed”) had *β* = −0.154, SE = 0.027, *z* = −5.704, 95% CI [−0.212, −0.106], supporting H3.

For H4, the chain mediation effect path (effect “bcd”) had *β* = −0.017, SE = 0.006, *z* = −2.833, 95% CI [−0.032, −0.007], supporting H4.

For H5, the moderating effect path (effect “h”) had *β* = 0.131, SE = 0.034, *z* = 3.853, 95% CI [0.063, 0.198], supporting H5.

For H7, the chain mediation plus moderation effect (effect “bchd”) had *β* = −0.002, SE = 0.001, *z* = −2.000, 95% CI [−0.005, −0.001], supporting H7.

#### 3.4.3 Effect analysis of model 2

Model 2 showed a total effect of −0.142, with SE = 0.049. The *z* value, calculated as Mean/SE, was 2.898, exceeding the 1.96 threshold, and the 95% confidence interval [−0.237, −0.043] did not include zero, indicating that the total effect was significant.

For H1, the direct effect path (effect “a”) had *β* = −0.117, SE = 0.041, *z* = −2.854, 95% CI [−0.200, −0.035], which did not include zero; the *z* value was significant, supporting H1.

For H2, the mediating effect path (effect “bf”) had *β* = −0.011, SE = 0.014, *z* = −0.764, 95% CI [−0.039, 0.016], indicating that H2 was not supported. From a statistical perspective, this non-significant mediation was mainly attributable to the non-significant path from SC to PA after EP and other model paths were simultaneously controlled. Although PSU significantly predicted SC, the indirect product term PSU → SC → PA was small in magnitude and its bootstrap confidence interval included zero, suggesting that SC did not independently transmit the effect of PSU on PA.

For H3, the mediating effect path (effect “ed”) had *β* = −0.154, SE = 0.027, z = −5.704, 95% CI [−0.212, −0.105], supporting H3.

For H4, the chain mediation effect path (effect “bcd”) had *β* = −0.018, SE = 0.006, *z* = −3.000, 95% CI [−0.033, −0.007], supporting H4.

For H6, the moderating effect path (effect “h”) had *β* = 0.119, SE = 0.032, *z* = 3.719, 95% CI [0.060, 0.186], supporting H6.

For H8, the chain mediation plus moderation effect (effect “bchd”) had *β* = −0.002, SE = 0.001, *z* = −2.000, 95% CI [−0.005, −0.001], supporting H8.

#### 3.4.4 Simple slope analysis of moderating effects

**Figure 3:**
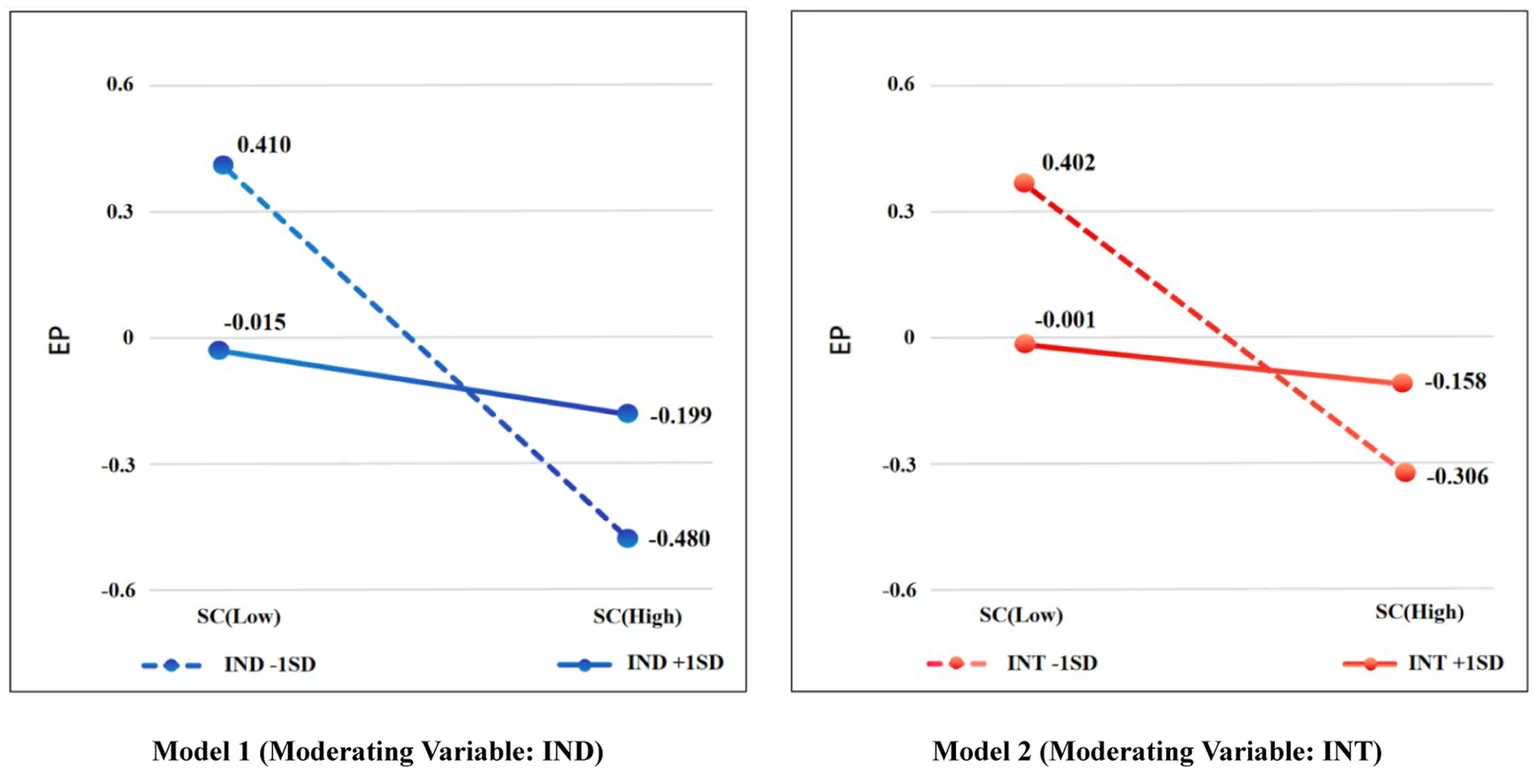
Simple slope plots. Simple slope plots of the moderating effects of IND and INT on the relationship between SC and EP. Low and high levels of SC and the moderators were defined as ±1 SD from the mean. The y-axis represents the predicted value of EP.

To further clarify the direction of the significant interaction effects, simple slope analyses were conducted separately for IND and INT, and revised slope plots were generated to improve readability (Figure 3). In the revised figure, the x-axis represents low and high levels of SC, the y-axis represents the predicted value of EP, and the lines represent low and high levels of the moderator defined as ±1 SD from the mean. Regression analyses were performed using PROCESS Model 1, and all relevant variables were mean-centered prior to analysis to reduce multicollinearity (Aiken et al., 1991; Hayes, 2017). In the analyses, EP was the dependent variable, SC was the independent variable, and IND and INT were tested as moderating variables. The predictive effect of SC on EP was examined at high and low levels of the moderating variables (±1 SD).

For Model 1, at low levels of IND (−1 SD), a strong negative relationship was observed between SC and EP. When SC was low, the predicted value of EP was 0.410; when SC was high, the predicted value decreased to −0.480, indicating that among individuals with low IND, higher SC was associated with substantially lower EP. In contrast, at high levels of IND (+1 SD), the predicted values were −0.015 for low SC and −0.199 for high SC, showing a smaller decrease and a flatter slope. This suggests that the negative predictive effect of SC on EP weakens as IND increases, further supporting H5.

For Model 2, at low levels of INT (−1 SD), a similarly strong negative relationship was observed between SC and EP. When SC was low, the predicted value of EP was 0.402; when SC was high, it decreased to −0.306, indicating a close association between increased SC and reduced EP among individuals with low INT. In contrast, at high levels of INT (+1 SD), the predicted values were −0.001 for low SC and −0.158 for high SC, showing a smaller decrease and a flatter slope. This indicates that the negative predictive effect of SC on EP also weakens as INT increases, further supporting H6.

## 4 Discussion

Based on data from 1,049 Chinese college students, this study examined the pathways and boundary conditions through which Problematic Smartphone Use (PSU) affects Physical Activity (PA). The results indicated that PSU significantly negatively predicted PA; however, the single mediating effect of Self-Control (SC) between PSU and PA was not significant. In contrast, Exercise Procrastination (EP) played a significant mediating role, and SC together with EP constituted a significant chain mediation pathway. Further analyses revealed that both Independent Self-Construal (IND) and Interdependent Self-Construal (INT) significantly moderated the relationship between SC and EP, and further moderated the chain mediation process through which PSU influenced PA via SC and EP.

### 4.1 Direct relationship between PSU and PA: a self-regulation imbalance perspective

PSU significantly negatively predicted college students’ PA, supporting H1. This finding indicates that the higher the level of PSU, the lower the PA, which is consistent with previous studies showing that PSU reduces PA, increases sedentary behavior, and promotes unhealthy behaviors (Yang et al., 2021; Zhu et al., 2023; Pirwani and Szabo, 2024).

Compared with prior studies that primarily explained this relationship from a time displacement or lifestyle perspective (Busch and McCarthy, 2021; Candussi et al., 2023), the present study further expands the explanation from a self-regulation imbalance perspective. PA requires planning, initiation, and persistence, while smartphone use provides immediate feedback, low-cost entertainment, and high stimulation, which easily satisfies instant gratification and emotion regulation needs (Hall and Fong, 2015). Therefore, PSU may not only occupy time for exercise but also weaken goal management and behavioral initiation, making it more difficult for students to translate exercise intentions into action. This finding suggests that insufficient PA among college students is not merely a time allocation issue, but may also reflect a self-regulation imbalance under digital lifestyle conditions.

### 4.2 Non-significant single mediation of SC: psychological resources do not necessarily translate directly into PA

Although PSU significantly negatively predicted SC, the single mediating effect of SC between PSU and PA was not significant, and H2 was not supported. This indicates that while PSU may impair SC, a reduction in SC does not necessarily directly lead to lower PA.

This result partially aligns with prior studies but also shows some differences. Previous research generally suggests that SC is a key psychological resource for maintaining healthy behaviors, enabling individuals to resist immediate temptations, pursue long-term goals, and engage in regular PA (de Ridder et al., 2012; Boat and Cooper, 2019; Andrade and Hoyle, 2023). This study agrees on the foundational role of SC in health behaviors, as results show that PSU significantly reduces SC. However, unlike previous research that treated SC as a direct determinant of PA, this study further finds that SC alone may not serve as a direct mediator connecting PSU to PA.

This finding suggests that PA is not solely determined by general psychological resources, but depends more on context-specific behavioral initiation, time management, and execution processes. In other words, PSU may first impair attentional control, impulse inhibition, and goal maintenance, but this depletion of psychological resources must further translate into delayed exercise planning, initiation difficulties, or behavioral avoidance before ultimately affecting PA. Thus, this study extends previous research by showing that the impact of PSU on PA is not simply a direct consequence of decreased SC, but rather a sequential process in which impaired SC leads to difficulties in exercise execution.

### 4.3 Mediating role of EP: behavioral execution mechanism from PSU to insufficient PA

EP played a significant mediating role between PSU and PA, supporting H3. Higher levels of PSU made students more likely to procrastinate on exercise, and higher levels of EP were associated with lower PA. This indicates that the influence of PSU on PA extends beyond time occupation or psychological resource depletion, translating into delays in exercise initiation and difficulties in behavioral execution.

This result aligns with prior research on EP, which is defined as the tendency to delay or avoid exercise behavior even when recognizing its importance or having an exercise plan (Kelly and Walton, 2021; Miao et al., 2024). Compared with general exercise intention or SC, EP more closely reflects the proximal behavioral manifestation of insufficient PA. This study further shows that in the context of PSU, students are easily attracted by smartphone entertainment, social media browsing, short videos, or games, reducing the priority of exercise behaviors and delaying planned PA.

Hence, this study advances prior research by identifying EP as a key behavioral mechanism. PSU may alter attention allocation and goal prioritization, leading to difficulties in exercise initiation and behavioral avoidance, ultimately reducing PA. This finding suggests that promoting PA among college students requires not only reducing PSU but also helping students overcome EP and improve the conversion of exercise intention into action.

### 4.4 Chain mediation of SC and EP: from psychological resource depletion to behavioral execution failure

SC and EP played a significant chain mediating role between PSU and PA, supporting H4. Specifically, higher levels of PSU were associated with lower SC; decreased SC further increased EP, ultimately reducing PA. This indicates that the effect of PSU on PA cannot be explained solely by a single psychological or behavioral factor, but reflects a sequential process from psychological resource depletion to behavioral execution difficulties.

This finding aligns with self-regulation theory, which emphasizes that SC is a crucial psychological resource for resisting immediate temptations, maintaining long-term goals, and regulating impulses (de Ridder et al., 2012; Andrade and Hoyle, 2023), while PA as a long-term health behavior requires sustained planning, initiation, and persistence (Hall and Fong, 2015). The results support the view that PSU may impair attentional control and goal maintenance, making it harder for individuals to disengage from immediate digital stimuli and allocate behavioral resources to PA.

Unlike prior studies that emphasized the direct effect of SC on health behaviors, this study further found that SC operates through EP as a proximal behavioral mechanism. That is, even when students know exercise is beneficial, impaired SC due to PSU increases the likelihood of delaying or avoiding exercise, ultimately reducing PA. This extends previous explanations of the relationship between PSU and PA, highlighting that insufficient PA may arise not only from time displacement or low exercise intention but also from increased EP following reduced SC. Theoretically, this finding suggests that self-regulation in PA should be understood not only as the possession of general psychological resources, but also as the successful translation of these resources into context-specific behavioral execution.

### 4.5 Moderating role of IND: autonomy as a boundary condition

IND significantly moderated the relationship between SC and EP, and further moderated the chain mediation path from PSU through SC and EP to PA, supporting H5 and H7. Simple slope analysis indicated that at low levels of IND, the negative predictive effect of SC on EP was stronger, whereas at high levels of IND, this effect was attenuated.

This finding is consistent with self-construal theory, which emphasizes autonomy, personal goals, and self-consistency (Markus and Kitayama, 1991; Singelis, 1994). Prior research suggests that individuals’ self-understanding influences goal setting, behavioral regulation, and health behavior choices (Cross et al., 2011). This study further shows that IND does not simply affect EP levels but alters the strength of SC’s effect on EP. Individuals with low IND lack stable autonomous goal support and thus rely more on SC to resist smartphone temptation and reduce procrastination, whereas individuals with high IND are more likely to view exercise as a personal goal and part of self-management, reducing dependence on general SC. Hence, IND serves as an important autonomy-related boundary condition in the effect of PSU on PA.

### 4.6 Moderating role of INT: social relationships as a boundary condition

INT significantly moderated the relationship between SC and EP, and further moderated the chain mediation path from PSU through SC and EP to PA, supporting H6 and H8. Simple slope analysis indicated that at low levels of INT, the negative predictive effect of SC on EP was stronger, whereas at high levels of INT, this effect was attenuated.

This finding also aligns with self-construal theory, which emphasizes social relationships, role responsibilities, and contextual adaptation (Markus and Kitayama, 1991; Cross et al., 2011). Prior studies suggest that interdependent individuals are more influenced by social norms, expectations, and relational contexts (Singelis, 1994; Cross et al., 2011). This study further shows that INT can buffer the impact of low SC on EP. Individuals with low INT rely less on peer support and group norms and thus depend more on personal SC, whereas high interdependent individuals may be supported by peer invitations, group atmosphere, and role responsibilities, so reduced SC does not immediately translate into procrastination. Therefore, INT represents a key social relationship boundary condition in the effect of PSU on PA.

### 4.7 Convergent moderating pattern of IND and INT

Although IND and INT represent different orientations of self-construal, the present findings showed a convergent moderating pattern: both IND and INT weakened the negative predictive effect of SC on EP and further moderated the chain mediation pathway from PSU to PA through SC and EP. This convergence suggests that self-construal may function as a broader psychological boundary condition in the self-regulation process (Markus and Kitayama, 1991; Singelis, 1994). Specifically, individuals with higher IND may rely more on autonomous goals, personal standards, and self-directed exercise intentions, whereas individuals with higher INT may benefit more from social expectations, peer support, and group norms (Cross et al., 2000; Gardner et al., 1999). Although these two orientations operate through different motivational sources, both may provide alternative regulatory resources that reduce excessive dependence on general SC when individuals face exercise procrastination (Lee et al., 2000). Therefore, the moderating roles of IND and INT should not be interpreted as opposite or mutually exclusive effects, but rather as two culturally meaningful pathways through which self-construal buffers the translation of reduced SC into EP (Singelis, 1994). This finding refines self-construal theory by suggesting that independent and interdependent orientations may provide different but functionally similar regulatory resources in the context of exercise behavior. Theoretically, this finding suggests that self-regulation in PA should be understood not only as the possession of general psychological resources, but also as the successful translation of these resources into context-specific behavioral execution.

## 5 Limitations and future research

Although this study constructed and validated a chain mediation and moderation model of the effect of PSU on college students’ PA, several limitations exist.

First, because this study adopted a cross-sectional design and measured all variables at a single time point, the findings should be interpreted as associations among variables rather than evidence of causal relationships. The study primarily relied on theoretical models and statistical results to interpret variable relationships, making it difficult to fully establish the temporal sequence among PSU, SC, EP, and PA. Future research could employ longitudinal designs, cross-lagged models, or intervention studies to further examine the dynamic changes and mechanisms among these variables.

Second, this study used convenience sampling, with participants recruited from universities in several provinces in China. Although the sample covered different regions and majors, potential sampling bias may still exist, and differences in region, university type, major distribution, and campus exercise environment may affect students’ smartphone use and PA behaviors. In addition, because the study was conducted in the Chinese cultural context, where independent and interdependent self-construal may coexist and jointly shape self-regulation and exercise behavior, the generalizability of the findings to other cultural contexts should be interpreted with caution. Future studies could include more representative samples and cross-cultural comparisons to further validate the model.

Third, the study relied primarily on self-report questionnaires, which may be affected by recall bias and social desirability. Although procedural controls and single-factor CFA suggested that severe common method bias was unlikely, the single-factor CFA should be regarded as a preliminary diagnostic approach and cannot fully eliminate concerns about common method variance. Residual common method bias cannot be completely ruled out because all core psychological variables were collected from the same respondents at the same time point. Such residual bias may have inflated the observed associations among psychological constructs, particularly the correlations between PSU and SC and between PSU and EP. Future research could incorporate objective measures such as smartphone screen time records, exercise app data, wearable devices, accelerometers, marker variables, temporal separation, or multi-source data to improve measurement accuracy and ecological validity.

## 6 Conclusion

This study, based on Chinese college students and grounded in self-regulation theory and self-construal theory, constructed and tested a chain mediation and moderation model linking PSU to PA. The results showed that PSU was significantly negatively associated with PA; the single mediating effect of SC was not significant; EP played a significant mediating role; and SC together with EP formed a significant chain mediation pathway.

Additionally, both IND and INT significantly moderated the relationship between SC and EP, and further influenced the chain mediation path through which PSU affected PA via SC and EP. Overall, PSU may be associated with lower PA through its links with lower SC and higher EP, with variations depending on individuals’ self-construal.

Practically, promoting PA among college students should not only focus on reducing smartphone use but also on enhancing SC, intervening in EP, establishing autonomous exercise goals, providing peer support, and creating a supportive campus exercise environment.

## Data Availability

The datasets presented in this study can be found in online repositories. The names of the repository/repositories and accession number(s) can be found in the article/Supplementary material.
