# Supplementary material for: Exploring the influence of problematic smartphone use on physical activity: chain mediation of Self-Control and Exercise Procrastination and moderation by Independent and Interdependent Self-Construal
Source: Front Psychol. 2026 Sep 3;17:1886306. doi: 10.3389/fpsyg.2026.1886306 (PMC13581811; doi:10.3389/fpsyg.2026.1886306)
Supplement: Supplementary file 1 [file Data_Sheet_1.PDF]

Supplementary Material S1

AMOS User-Defined Estimands: Parameter Mapping and VB.NET Source Code

For: Exploring the Influence of Problematic Smartphone Use on Physical Activity: Chain Mediation of Self-Control and Exercise Procrastination and Moderation by Independent and Interdependent Self-Construal

Purpose. This supplement documents the exact parameter-to-path mapping and the VB.NET implementation used to define the hypothesis-specific estimands reported in Tables 5 and 6. The procedure uses the general User-Defined Estimands capability of IBM SPSS Amos 31.0 and is implemented in the Amos Program Editor. It is not a third-party plugin or a separate SEM estimator.

S1.1 Software and implementation

Software: IBM SPSS Amos 31.0. The estimands were implemented as a Visual Basic .NET (VB.NET) program in the Amos Program Editor. The script accesses labelled structural parameters using the Amos ParameterValue() function. For each bootstrap sample, the script returns the user-defined functions of the labelled model parameters. Bootstrap standard errors and bias-corrected confidence intervals were obtained from 5,000 resamples. Statistical significance was evaluated primarily by whether the 95% bootstrap confidence interval excluded zero.

S1.2 Parameter-to-path mapping

Parameter	Model 1 (IND)	Model 2 (INT)	Interpretation
a	PSU → PA	PSU → PA	Direct path from PSU to PA
b	PSU → SC	PSU → SC	PSU to self-control
c	SC → EP	SC → EP	Self-control to exercise procrastination
d	EP → PA	EP → PA	Exercise procrastination to physical activity
e	PSU → EP	PSU → EP	PSU to exercise procrastination
f	SC → PA	SC → PA	Self-control to physical activity
g	IND → EP	INT → EP	Moderator main effect on EP
h	SC × IND → EP	SC × INT → EP	Interaction/moderation effect on EP

S1.3 User-defined estimands

Output label	Formula	Meaning
a	a	Direct effect
bf	b × f	Indirect effect through SC
ed	e × d	Indirect effect through EP
bcd	b × c × d	Serial mediation effect through SC and EP
g	g	Moderator main effect
h	h	Interaction/moderation effect
bchd	b × c × h × d	Moderated serial mediation estimand as specified in the manuscript
total	a + bf + ed + bcd + g + h + bchd	Aggregate sum of the hypothesis-specific effects reported in Tables 5 and 6

S1.4 Complete VB.NET source code

The following code reproduces the estimand definitions above. The same source code can be used for both models provided that the AMOS path parameters are labelled a–h exactly as shown in Section S1.2.

```
#Region "Header"
Option Explicit On
Option Strict On
Imports System
Imports Microsoft.VisualBasic
Imports AmosEngineLib
Imports AmosEngineLib.AmosEngine
Imports AmosEngineLib.AmosEngine.TMatrixID
Imports MiscAmosTypes
Imports MiscAmosTypes.cDatabaseFormat
#End Region

Public Class CUserValue : Implements IUserValue

    Function Value(groupNumber As Integer, _
                bootstrapSampleNumber As Integer, _
                v As CValue) As Object Implements IUserValue.Value

        Dim x(7) As Double

        ' H1: direct effect
        x(0) = v.ParameterValue("a")

        ' H2: indirect effect through SC
        x(1) = v.ParameterValue("b") * v.ParameterValue("f")

        ' H3: indirect effect through EP
        x(2) = v.ParameterValue("e") * v.ParameterValue("d")

        ' H4: serial mediation effect
        x(3) = v.ParameterValue("b") * v.ParameterValue("c") * v.ParameterValue("d")

        ' Moderator main effect (IND in Model 1; INT in Model 2)
        x(4) = v.ParameterValue("g")

        ' H5/H6: interaction (moderation) effect
        x(5) = v.ParameterValue("h")

        ' H7/H8: moderated serial mediation estimand
        x(6) = v.ParameterValue("b") * v.ParameterValue("c") * v.ParameterValue("h") * v.ParameterValue("d")

        ' Aggregate sum reported in Tables 5 and 6
        x(7) = x(0) + x(1) + x(2) + x(3) + x(4) + x(5) + x(6)

        Return x
    End Function

    Function Label(groupNumber As Integer) As Object Implements IUserValue.Label

        Dim labels(7) As String

        labels(0) = "a"
        labels(1) = "bf"
        labels(2) = "ed"
        labels(3) = "bcd"
        labels(4) = "g"
        labels(5) = "h"
        labels(6) = "bchd"
```

```
        labels(7) = "total"

        Return labels
    End Function

    Public Sub Initialize() Implements IUserValue.Initialize
    End Sub

End Class
```

S1.5 Reproduction procedure

1. In each AMOS model, assign the path parameter labels a–h exactly as listed in Section S1.2.

2. Open the IBM SPSS Amos 31 Program Editor and use the general user-defined estimand template (VB.NET).

3. Paste the source code in Section S1.4, verify that the parameter labels match the AMOS model, and check the syntax.

4. In AMOS Analysis Properties, enable bootstrap estimation with 5,000 resamples and bias-corrected confidence intervals.

5. Calculate estimates and inspect the User-defined estimands output, including the point estimates, bootstrap standard errors, confidence intervals, and significance results.

6. For Model 1, interpret g as IND → EP and h as SC × IND → EP. For Model 2, interpret g as INT → EP and h as SC × INT → EP.

S1.6 Reference

Arbuckle, J. L. (2025). *IBM SPSS Amos 31 User's Guide*. IBM Corporation. https://www.ibm.com/support/pages/ibm-spss-amos-31-documentation
