# Supplementary material for: Exploring the influence of problematic smartphone use on physical activity: chain mediation of Self-Control and Exercise Procrastination and moderation by Independent and Interdependent Self-Construal
Source: Front Psychol. 2026 Sep 3;17:1886306. doi: 10.3389/fpsyg.2026.1886306 (PMC13581811; doi:10.3389/fpsyg.2026.1886306)
Supplement: Supplementary file 2 [file Table_1.DOCX]

| **1. PSU questionnaire (1 = Strongly disagree, 6 = Strongly agree)** | |
| --- | --- |
| Item No. | Item |
| 1 | Missing planned work due to smartphone use. |
| 2 | Having difficulty concentrating in class, or when working or completing tasks due to smartphone use. |
| 3 | Feeling pain in the wrist or back of the neck while using a smartphone. |
| 4 | Being unable to tolerate not having a smartphone. |
| 5 | Feeling impatient and irritated when I am not holding my smartphone. |
| 6 | Thinking about my smartphone even when I am not using it. |
| 7 | I would never give up using my smartphone even if my daily life were greatly affected by it. |
| 8 | Constantly checking my smartphone so as not to miss conversations among others on social networking apps such as WeChat. |
| 9 | Using my smartphone longer than I had intended. |
| 10 | People around me tell me that I use my smartphone too much. |

| **SC questionnaire（1 = Strongly disagree, 5 = Strongly agree）** | |
| --- | --- |
| Item No. | Item |
| 1 | I am good at resisting temptation. |
| 2 | I am able to get rid of bad habits. |
| 3 | I am hardworking. |
| 4 | I am able to concentrate. |
| 5 | Even if something is enjoyable but bad for me, I can control myself and avoid doing it. |
| 6 | I have strong self-discipline. |
| 7 | Pleasure and entertainment do not prevent me from completing my work. |
| 8 | I usually speak in an appropriate manner. |
| 9 | I am able to work effectively toward long-term goals. |
| 10 | Even when something is tempting, I can stop myself from doing the wrong thing. |
| 11 | I often carefully consider various options before taking action |

| **EP questionnaire（1 = Strongly disagree, 5 = Strongly agree）** | |
| --- | --- |
| Item No. | Item |
| 1 | I often delay my planned exercise for too long, which causes unnecessary negative effects on my physical and mental state or daily efficiency. |
| 2 | When I have an exercise task to complete, I will prioritize completing the exercise before dealing with other less important matters. |
| 3 | My life would be better if I could start exercising earlier. |
| 4 | When I should be exercising, I often turn to doing other things instead. |
| 5 | When reviewing my daily schedule, I realize that I could have used my time better for exercise. |
| 6 | I am able to arrange my time reasonably and efficiently for exercise. |
| 7 | The extent to which I procrastinate on exercise has exceeded a reasonable range. |
| 8 | I often procrastinate on exercise. |
| 9 | As long as I think I should exercise, I will take action immediately. |

| **IPAQ-SF** | |
| --- | --- |
| Item No. | Item |
| 1 | During the last 7 days, on how many days did you do vigorous physical activities? |
| 2 | How much time did you usually spend doing vigorous physical activities on one of those days? |
| 3 | During the last 7 days, on how many days did you do moderate physical activities? |
| 4 | How much time did you usually spend doing moderate physical activities on one of those days? |
| 5 | During the last 7 days, on how many days did you walk for at least 10 minutes at a time? |
| 6 | How much time did you usually spend walking on one of those days? |
| 7 | During the last 7 days, how much time did you usually spend sitting on a weekday? |

| **IND questionnaire（1 = Completely disagree, 7 = Completely agree）** | | |
| --- | --- | --- |
| Item No. | Item | |
| 1 | I would rather express my thoughts directly than be misunderstood. | |
| 2 | Speaking up in class is not a problem for me. | |
| 3 | It is important for me to maintain an active imagination. | |
| 4 | I feel comfortable when I am praised or rewarded individually. | |
| 5 | I respect people who are modest and low-key. | |
| 6 | I behave consistently at home and at school. | |
| 7 | For me, my main concern is being able to take care of myself. | |
| 8 | When meeting someone I have only recently become acquainted with, I naturally call them by their first name, even if they are much older than me. | |
| 9 | When interacting with people I have just met, I prefer to be direct. | |
| 10 | I am willing to be different from others in many ways. | |
| 11 | Having a personality that is independent of others is very important to me. | |
| 12 | I believe that health is the most important thing. | |
| **INT questionnaire（1 = Completely disagree, 7 = Completely agree）** | | |
| Item No. | Item | |
| 1 | I respect people in authority with whom I interact. | |
| 2 | Maintaining a harmonious relationship with others is very important to me. | |
| 3 | The happiness of people around me is my happiness. | |
| 4 | For the benefit of the group, I would sacrifice my own interests. | |
| 5 | I often feel that maintaining good interpersonal relationships is more important than my own achievements. | |
| 6 | My behavior is consistent no matter whom I am with. | |
| 7 | When making plans for my education or career, I should consider my parents’ suggestions. | |
| 8 | Respecting group decisions is the most important thing to me. | |
| 9 | If the group I belong to needs me, I will stay there even if I am not happy. | |
| 10 | If a friend encounters difficulties, I feel responsible for helping him or her. | |
| 11 | Even if my opinions differ from those of group members, I avoid arguments. | |
| 12 | I will voluntarily give up my seat to my teacher on public transportation. | |
| **1. What is your gender?** | |  |
| ① Male | |  |
| ② Female | |  |
| **2. What is your current year of study?** | |  |
| ① First year | |  |
| ② Second year | |  |
| ③ Third year | |  |
| ④ Fourth year | |  |
| ⑤ Fifth year or above / Delayed graduation | |  |
| **3. What is your major category?** | |  |
| ① Humanities and Social Sciences | |  |
| ② Natural Sciences and Engineering | |  |
| ③ Physical Education | |  |
| ④ Health and Medicine | |  |
| ⑤ Education | |  |
| ⑥ Agriculture | |  |
| **4. How would you rate your subjective health status?** | |  |
| ① Very poor | |  |
| ② Poor | |  |
| ③ Fair | |  |
| ④ Good | |  |
| ⑤ Very good | |  |
